# Physiological mechanisms behind respiratory variations in right atrial pressure in pulmonary hypertension

**DOI:** 10.1038/s41598-024-61825-6

**Published:** 2024-05-31

**Authors:** Athiththan Yogeswaran, Bruno Brito da Rocha, Zvonimir A. Rako, Samuel J. Kaufmann, Simon Schäfer, Nils Kremer, Hossein Ardeschir Ghofrani, Werner Seeger, Khodr Tello

**Affiliations:** 1 Department of Internal Medicine, Universities of Giessen and Marburg Lung Center (UGMLC), Institute for Lung Health (ILH), Cardio-Pulmonary Institute (CPI), Member of the German Center for Lung Research (DZL), Giessen, Germany; 2 Department of Pneumology, Kerckhoff Heart, Rheuma and Thoracic Center, Bad Nauheim, Germany; 3 Department of Medicine, Imperial College London, London, UK; 4 Department of Internal Medicine, Justus-Liebig-University Giessen, Klinikstrasse 32, 35392 Giessen, Germany

**Keywords:** Conductance catheterization, Diastolic dysfunction, End-diastolic elastance, Exercise haemodynamics, Prognosis, Pulmonary hypertension, Respiratory variation, Retrospective analysis, Right atrial pressure, Right ventricular dysfunction, Physiology, Cardiology, Medical research, Pathogenesis

## Abstract

Impaired respiratory variation of right atrial pressure (RAP) in severe pulmonary hypertension (PH) suggests difficulty tolerating increased preload during inspiration. Our study explores whether this impairment links to specific factors: right ventricular (RV) diastolic function, elevated RV afterload, systolic RV function, or RV-pulmonary arterial (PA) coupling. We retrospectively evaluated respiratory RAP variation in all participants enrolled in the EXERTION study. Impaired respiratory variation was defined as end-expiratory RAP − end-inspiratory RAP ≤ 2 mm Hg. RV function and afterload were evaluated using conductance catheterization. Impaired diastolic RV function was defined as end-diastolic elastance (Eed) ≥ median (0.19 mm Hg/mL). Seventy-five patients were included; PH was diagnosed in 57 patients and invasively excluded in 18 patients. Of the 75 patients, 31 (41%) had impaired RAP variation, which was linked with impaired RV systolic function and RV-PA coupling and increased tricuspid regurgitation and Eed as compared to patients with preserved RAP variation. In backward regression, RAP variation associated only with Eed. RAP variation but not simple RAP identified impaired diastolic RV function (area under the receiver operating characteristic curve [95% confidence interval]: 0.712 [0.592, 0.832] and 0.496 [0.358, 0.634], respectively). During exercise, patients with impaired RAP variation experienced greater RV dilatation and reduced diastolic reserve and cardiac output/index compared with patients with preserved RAP variation. Preserved RAP variation was associated with a better prognosis than impaired RAP variation based on the 2022 European Society of Cardiology/European Respiratory Society risk score (chi-square *P* = 0.025) and survival free from clinical worsening (91% vs 71% at 1 year and 79% vs 50% at 2 years [log-rank *P* = 0.020]; hazard ratio: 0.397 [95% confidence interval: 0.178, 0.884]). Subgroup analyses in patients with group 1 and group 4 PH demonstrated consistent findings with those observed in the overall study cohort. Respiratory RAP variations reflect RV diastolic function, are independent of RV-PA coupling or tricuspid regurgitation, are associated with exercise-induced haemodynamic changes, and are prognostic in PH.

**Trial registration.** NCT04663217.

## Introduction

Pulmonary hypertension (PH) is a chronic disease characterized by narrowing of pulmonary vascular lumen and subsequently increased right ventricular (RV) afterload^1^. Consequently, the right ventricle undergoes (mal-)adaptations driven by the burden of both pressure and volume overload^2^. This process results in the development of pathophysiological features including secondary tricuspid regurgitation and impaired RV diastolic and systolic function as the disease progresses^2^.

Impaired RV function is associated with elevated RV filling pressures, which have already been described in patients with PH as well as other diseases^1^. In 1873, Kussmaul published an important clinical observation: patients with compromised cardiac filling due to conditions such as constrictive pericarditis or restrictive cardiomyopathy had a paradoxical increase in jugular venous pressure during inspiration compared with healthy individuals^3^. In an intriguing parallel, this phenomenon extends to patients with right-sided heart failure and PH. Within this context, researchers have shown that impaired respiratory variability of right atrial pressure (RAP) in PH is an indicator of disease severity and right-sided heart function^4^. However, the mechanisms behind this phenomenon in patients with PH have not been fully investigated. Plausible factors include tricuspid regurgitation, RV systolic dysfunction, and RV diastolic dysfunction—each with the potential to increase RV filling pressure and to impede the variation of RAP during spontaneous inspiration. Since assessment of RV diastolic function and RV-pulmonary arterial (PA) coupling is technically challenging, studies on this topic were lacking. To address this knowledge gap comprehensively, our study utilizes gold-standard assessments of RV diastolic and systolic function via pressure–volume loop analysis to investigate the physiological mechanisms behind and functional consequences of respiratory variations in RAP.

## Methods

### Study design and population

All patients enrolled in the prospectively recruiting *Exercise Hemodynamic, Right Ventricular Coupling and Echocardiography in Pulmonary Hypertension* (EXERTION) study (ClinicalTrials.gov identifier: NCT04663217) from 2020 to 2022 were included in this post-hoc analysis. Diagnosis of PH was made by a multidisciplinary board following contemporary guidelines^1^. Control patients were initially referred due to suspected PH and exertional dyspnoea; PH was ruled out through invasive diagnostics (mean pulmonary artery pressure at rest ≤ 20 mm Hg). Among other criteria, severe lung disease was an important exclusion criterion due to its potential impact on RAP variation. Additional inclusion and exclusion criteria are detailed for the EXERTION study (NCT04663217). The study adheres to the principles of the Declaration of Helsinki and was approved by the local Ethics Committee of the University of Giessen Medical Faculty (approval number 117/16). Written informed consent was obtained from all participating patients.

### Right heart catheterization and assessment of RAP variation during spontaneous inspiration

Right heart catheterization was performed as previously described^5^. Briefly, under sonographic guidance and local anaesthesia, a 7F sheath was introduced into the internal jugular vein. Measurements of pulmonary artery wedge pressure and mean pulmonary artery pressure were acquired during end-expiratory phases using a Swan-Ganz catheter. RAP was evaluated as the mean over several cardiac cycles during spontaneous respiration. Respiratory variation was computed as the disparity between end-expiratory and end-inspiratory RAP values (ΔRAP)^4^. A ΔRAP greater than 2 mm Hg indicated preserved respiratory variation^4^. Cardiac output and index were derived through the direct Fick method, when applicable, or by utilizing thermodilution^1^.

### Pressure–volume loop assessment

Pressure–volume loops were obtained using conductance catheterization as described previously^6^. A 4F pressure–volume catheter (CA-41063; CD Leycom, Zoetermeer, The Netherlands) was positioned in the RV apex under sonographic guidance, and an intracardiac analyser (Inca; CD Leycom) was utilized to visualize real-time pressure–volume loops. The calculation of arterial elastance (Ea) involved determining the ratio of end-systolic pressure to stroke volume, and end-systolic elastance (Ees) was computed using the RV single-beat approach^7^. RV-PA coupling was defined as the Ees/Ea ratio. Diastolic stiffness (β) was determined using a custom MATLAB programme and fitting a nonlinear exponential curve through three data points on the diastolic section of the pressure–volume loops^8^. Additionally, we determined Eed by applying the relationship ${\text{dP}}/{\text{dV}}\, = \,\alpha \beta \, \times \,{\text{e}}^{{(\beta \, \times \,{\text{EDV}})}}$ at calculated end-diastolic volumes (EDV), with α representing a curve-fit parameter^9^.

### Exercise protocol

Patients underwent exercise in a semi-supine position after the placement of the conductance catheter, continuing until exhaustion. We followed an incremental protocol, increasing the workload by 20 W every 2–4 min. For patients unable to begin at a 20-W workload, we adjusted the initial workload to a minimum of 5 W, with subsequent 5-W increments every 2–4 min until exhaustion. The exercise session had a maximum duration of 10–12 min. Simultaneously, we conducted echocardiography alongside symptom assessments. Pressure–volume loops were determined at two specific time points: before exercise (baseline) and at the point of maximum exertion.

### Data assessment and statistical analyses

Adherence to normal distribution of all variables was assessed using the Shapiro–Wilk test. For normally distributed parameters, we present the mean ± standard deviation and employed (pairwise) t-tests for comparing means between groups. Non-normally distributed parameters are displayed as medians [Q1, Q3], and the Wilcoxon rank sum test was utilized for comparing medians across groups. Categorical parameters were compared by the chi-square test. Spearman's rho coefficient was used for correlation analyses. ∆RAP was utilized as continuous parameter for correlation analyses. The *pROC* package was used for receiver operating characteristic (ROC) analyses. Clinical worsening was assessed in August 2023 for all patients included in the study. Clinical worsening was defined as meeting at least one of the following criteria: (1) 6-min walk distance (6MWD) decrease ≥ 15% in two consecutive tests; (2) worsening of World Health Organization (WHO) functional class; (3) hospitalization; (4) escalation of diuretics (either dose increase or additional diuretics); (5) escalation of PH treatment; (6) lung transplantation; and (7) death. Survival analyses included Kaplan–Meier and univariate Cox regression analyses. Backward regression analysis was performed to ascertain the relationship between RAP variation and other parameters. All statistical procedures were performed using R version 4.0.4 (The R Foundation, Vienna).

### Ethics approval and consent to participate

The study adheres to the principles of the Declaration of Helsinki and was approved by the local Ethics Committee of the University of Giessen Medical Faculty (approval number 117/16). Written informed consent was obtained from all participating patients.

## Results

### Patient characteristics

A total of 75 patients were enrolled, with 64% being female. The median age was 69 [58, 76] years, and most of the patients experienced significant exertional dyspnoea (64% were classified as WHO functional class III or IV). Of the 75 patients, 25 (33%) were diagnosed with group 1 PH, while 12 (16%) and 20 (27%) were classified as group 2 and group 4, respectively. Additionally, 18 patients (24%) had PH invasively ruled out. Table 1 shows impaired pulmonary haemodynamics in the overall study population.

**Table 1:** Patient characteristics.

	n	RAP variation ≤ 2 mm Hg (n = 31)	RAP variation > 2 mm Hg (n = 44)	Combined (n = 75)	*P*
Age, years	75	70.0 [60.0, 78.0]	66.5 [53.2, 73.0]	69.0 [57.5, 76.0]	0.13^a^
Female sex, n (%)	75	20 (65)	28 (64)	48 (64)	0.938^b^
BMI, kg/m^2^	75	24.53 [23.20, 29.57]	29.74 [25.87, 33.41]	27.73 [23.66, 32.16]	0.004^a^
Diagnosis, n (%)	75				
Control		5 (16)	13 (30)	18 (24)	0.206^b^
Group 1		10 (32)	15 (34)	25 (33)	
Group 2		8 (26)	4 (9)	12 (16)	
Group 4		8 (26)	12 (27)	20 (27)	
WHO FC, n (%)	75				0.057^b^
I		1 (3)	1 (2)	2 (3)	
II		5 (16)	15 (34)	20 (27)	
III		24 (77)	20 (45)	44 (59)	
IV		0 (0)	4 (9)	4 (5)	
NA		1 (3)	4 (9)	5 (7)	
6MWD, m	26	375 [275, 480]	400 [332, 439]	400 [304, 450]	0.743^a^
VO_2_max, mL/kg/min	47	12.25 [10.47, 14.67]	13.80 [11.85, 17.50]	13.00 [11.20, 17.50]	0.236^a^
BNP, pg/mL	74	243.0 [68.0, 417.0]	46.0 [16.0, 119.0]	80.5 [26.0, 238.0]	< 0.001^a^
TAPSE, mm	72	19.4 [14.3, 22.2]	21.6 [19.6, 23.6]	21.2 [18.3, 22.8]	0.01^a^
RA ESA, cm^2^	75	21.70 [14.35, 33.25]	16.45 [13.20, 18.97]	17.70 [13.65, 23.20]	0.01^a^
RV EDD, mm	75	46.7 ± 10.8	43.1 ± 6.7	44.6 ± 8.8	0.117^c^
TAPSE/PASP, mm/mm Hg	66	0.272 [0.219, 0.490]	0.474 [0.294, 0.741]	0.399 [0.252, 0.594]	0.006^a^
RV strain, %	75	− 16.6 ± 5.8	− 19.8 ± 3.4	− 18.5 ± 4.8	0.007^c^
RA reservoir strain, %	74	24.6 ± 15.1	36.7 ± 12.6	31.8 ± 14.8	< 0.001^c^
RA conduit strain, %	74	− 12.7 ± 6.8	− 17.4 ± 9.3	− 15.5 ± 8.7	0.015^c^
RA contractile strain, %	74	− 8.9 [− 18.2, − 2.0]	− 19.2 [− 23.2, − 12.7]	− 17.1 [− 22.5, − 8.5]	0.002^a^
3D RV EDV, mL	75	130.0 ± 43.3	124.4 ± 41.1	126.7 ± 41.8	0.578^c^
TI, n (%)	75				0.009^b^
None		2 (6)	9 (20)	11 (15)	
Mild		6 (19)	18 (41)	24 (32)	
Moderate		16 (52)	11 (25)	27 (36)	
Severe		5 (16)	1 (2)	6 (8)	
NA		2 (6)	5 (11)	7 (9)	
IVC diameter, mm	73	21.1 ± 6.0	17.7 ± 3.7	19.1 ± 5.1	0.008^c^
RAP, mm Hg	68	7.0 [4.8, 11.0]	6.0 [5.0, 9.0]	6.0 [5.0, 9.0]	0.254^a^
mPAP, mm Hg	74	37.0 [25.5, 46.5]	28.0 [19.0, 39.0]	35.0 [20.2, 43.5]	0.124^a^
TPR, WU	74	7.20 [5.11, 10.25]	5.56 [3.19, 8.64]	6.08 [3.76, 9.31]	0.04^a^
PAWP, mm Hg	74	10.0 [8.0, 14.5]	10.0 [7.0, 12.0]	10.0 [7.0, 12.8]	0.253^a^
Cardiac index, L/min/m^2^	74	2.61 ± 0.63	2.76 ± 0.56	2.70 ± 0.59	0.275^c^
PVR, WU	74	4.59 [2.35, 7.45]	3.09 [1.69, 6.29]	3.70 [1.90, 6.83]	0.164^a^
SvO_2_, %	74	65.8 [61.4, 68.7]	68.3 [66.1, 71.7]	67. 5 [63.3, 70.6]	0.009^a^
Eed, mm Hg/mL	75	0.272 [0.179, 0.364]	0.151 [0.108, 0.228]	0.188 [0.129, 0.283]	< 0.001^a^
Ees, mm Hg/mL	75	0.748 [0.682, 0.995]	0.613 [0.408, 0.838]	0.696 [0.520, 0.944]	0.017^a^
Ea, mm Hg/mL	75	0.772 [0.571, 1.045]	0.457 [0.318, 0.759]	0.579 [0.398, 0.905]	< 0.001^a^
Ees/Ea	75	1.042 [0.813, 1.232]	1.349 [1.011, 1.548]	1.164 [0.946, 1.457]	0.003^a^
ESC/ERS risk, n (%)	75				0.025^b^
1		11 (35)	29 (66)	40 (53)	
2		17 (55)	14 (32)	31 (41)	
3		3 (10)	1 (2)	4 (5)	
S′/RAAi, m^2^/(s·cm)	75	1.042 ± 0.644	1.424 ± 0.524	1.266 ± 0.603	0.009^c^
Respiratory RAP variation, mm Hg	75	1.0 [0.8, 1.3]	4.7 [3.4, 6.0]	3.0 [1.1, 5.1]	< 0.001^a^

Data are presented as n (%) for categorical variables, mean ± standard deviation for normally distributed continuous variables, and median [Q1, Q3] for non-normally distributed continuous variables.

6MWD, 6-min walk distance; BMI,  body mass index; BNP,  brain natriuretic peptide; Ea,  arterial elastance; EDD,  end-diastolic diameter; EDV,  end-diastolic volume; Eed,  end-diastolic elastance; Ees,  end-systolic elastance; ESA,  end-systolic area; ESC/ERS,  European Society of Cardiology/European Respiratory Society; IVC,  inferior vena cava; mPAP,  mean pulmonary artery pressure; NA,  not available; PASP,  pulmonary artery systolic pressure; PAWP,  pulmonary artery wedge pressure; PVR,  pulmonary vascular resistance; RA,  right atrial; RAP,  right atrial pressure; RV,  right ventricular; S′/RAAi,  ratio of peak lateral tricuspid annulus systolic velocity to right atrial area index; SvO_2_,  mixed venous oxygen saturation; TAPSE = tricuspid annular plane systolic excursion; TI,  tricuspid insufficiency; TPR,  total pulmonary resistance; VO_2_max,  maximum oxygen uptake; WHO FC,  World Health Organization functional class; WU,  Wood Units.

^a^Wilcoxon test.

^b^Chi-square test.

^c^T-test.

The median RAP variation during spontaneous breathing was 3 [1, 5] mm Hg, with 31 patients (41%) exhibiting impaired RAP variation defined as ΔRAP ≤ 2 mm Hg. Interestingly, patients with impaired RAP variation displayed lower body mass index, higher brain natriuretic peptide levels, and impaired echocardiographic RV and right atrial (RA) function compared with patients with preserved RAP variation (Table 1). Exercise capacity (maximum oxygen uptake, 6MWD) was numerically but not statistically significantly lower in the patients with impaired RAP variation. Importantly, both RV-PA coupling (Ees/Ea) and RV diastolic function (Eed) were significantly impaired at rest in these patients. TAPSE/PASP, as a surrogate marker of RV-PA coupling, exhibited a significant impairment as well (Table 1).

### Physiological basis of RAP variation

**Figure 1:**
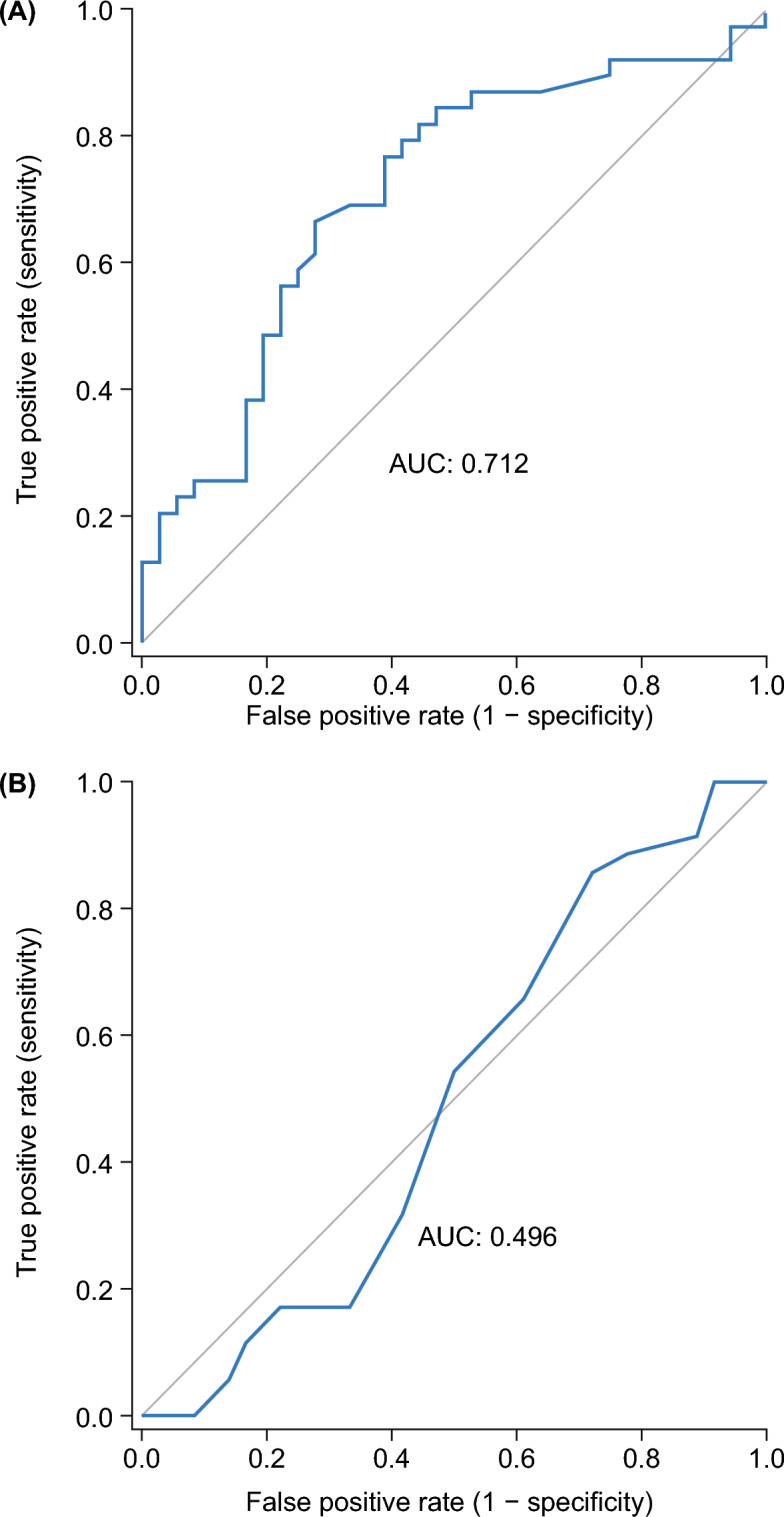
ROC analysis of (**A**), respiratory RAP variation and (**B**), RAP for prediction of diastolic dysfunction. Diastolic dysfunction was defined as end-diastolic elastance ≥ median (= 0.19 mm Hg/mL). RAP, right atrial pressure; ROC, receiver operating characteristic.

Correlation analyses were conducted to examine the relationship between RAP variation and RV diastolic function, load-independent systolic function, and RV-PA coupling. A significant correlation between ΔRAP and Eed was observed (Spearman’s rho =  − 0.41, *P* < 0.001), whereas no correlation was evident between ΔRAP and Ees (Spearman’s rho =  − 0.21, *P* = 0.07) or between ΔRAP and Ees/Ea (Spearman’s rho = 0.21, *P* = 0.08). Consequently, ΔRAP effectively identified impaired diastolic RV function (defined as Eed ≥ median [= 0.19 mm Hg/mL]) with a good area under the ROC curve (0.712 [95% confidence interval: 0.592, 0.832], Fig. 1A). By contrast, the simple assessment of RAP could not identify impaired RV diastolic function, as shown in Fig. 1B (area under the ROC curve: 0.496 [95% confidence interval: 0.358, 0.634]). Through regression analysis, we determined that only Eed, but not Ees, Ea or tricuspid regurgitation severity, showed a significant association with ΔRAP (Table 2).

**Table 2:** Regression analysis of the association of RV function and afterload with respiratory RAP variation.

	Estimate	Standard error	t value	*P*
(Intercept)	5.0288	0.7761	6.479	< 0.001
End-diastolic elastance	− 7.0235	2.6227	− 2.678	0.009
End-systolic elastance	0.3810	1.3192	0.289	0.774
Arterial elastance	− 0.2087	1.2323	− 0.169	0.866
Tricuspid insufficiency	− 0.1583	0.3590	− 0.441	0.661

RAP,  right atrial pressure; RV,  right ventricular.

Notably, during exercise, there was no difference in contractile reserve (measured as the change in Ees from rest to exercise) between the impaired and preserved RAP variation groups (0.20 [0.01, 0.35] mm Hg/mL and 0.23 [0.03, 0.44] mm Hg/mL, respectively; *P* = 0.42). Consistent with this finding, the ΔEes/Ea ratio was also comparable between the two groups (− 0.05 [− 0.21, 0.13] and − 0.01 [− 0.38, 0.25], respectively; *P* = 0.96). However, RV dilatation (measured as the change in RV EDV from rest to exercise) was more pronounced in patients with impaired RAP variation than in those with preserved RAP variation (29 [8, 40] mL vs 3 [− 7, 24] mL, *P* = 0.002). RV diastolic function during exertion was significantly impaired in patients with impaired RAP variation (RV end-diastolic pressure: 16 [10, 25] mm Hg vs 12 [7, 17] mm Hg, *P* = 0.049; Eed: 0.36 [0.28, 0.51] mm Hg/mL vs 0.27 [0.12, 0.45] mm Hg/mL, *P* = 0.012). Concordantly, both peak cardiac output and cardiac index were significantly reduced in the impaired RAP variation group (cardiac output: 6.4 [4.7, 8.3] L/min vs 7.7 [6.2, 10.2] L/min, *P* = 0.015; cardiac index: 3.2 [2.6, 4.4] L/min/m^2^ vs 4.2 [3.3, 4.8] L/min/m^2^, *P* = 0.031).

### Prognostic significance of RAP variation

**Figure 2:**
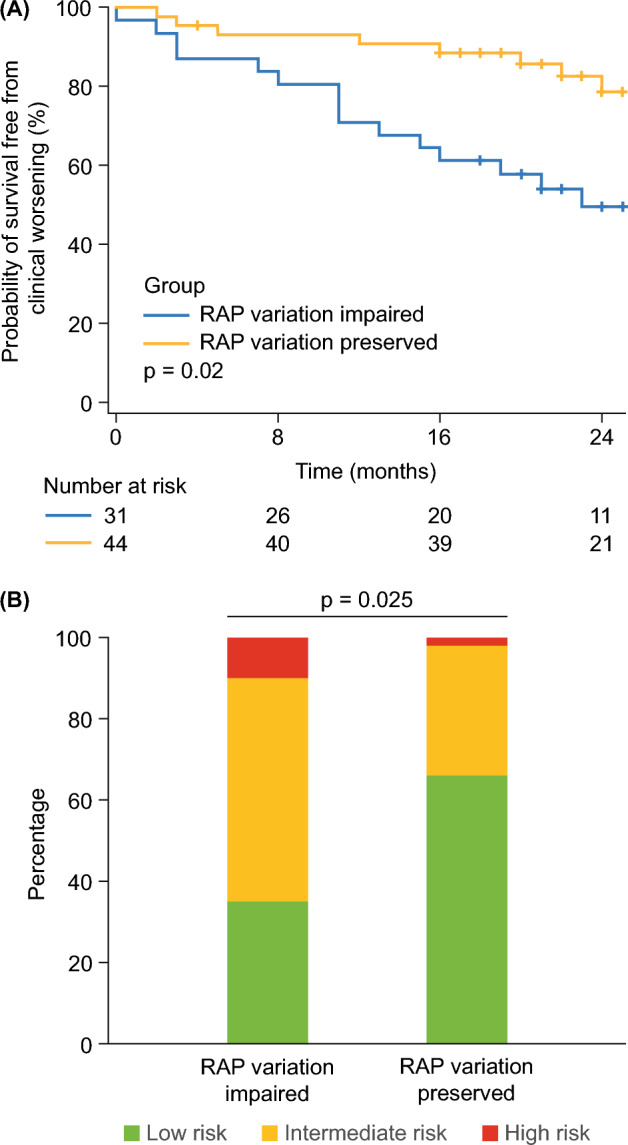
Prognostic relevance of respiratory RAP variation. (**A**) Time to clinical worsening (Kaplan–Meier analysis) and (**B**) European Society of Cardiology/European Respiratory Society risk distribution were compared between patients with impaired (≤ 2 mm Hg) and preserved (> 2 mm Hg) RAP variation. RAP, right atrial pressure.

Lastly, we explored the relationship between impaired respiratory variation of RAP and clinical worsening. Overall, clinical worsening occurred in 25 patients(33%) (escalation of PH-targeted therapy in eight cases [32%], increase in diuretics in five cases [20%], hospitalization in four cases [16%], substantial decrease of 6MWD in four cases [16%], worsening of WHO functional class in two cases [8%], and death in two cases [8%]). As depicted in Fig. 2A, patients with preserved RAP variation during spontaneous breathing exhibited significantly higher survival free from clinical worsening than patients with impaired RAP variation (worsening-free survival at 1 and 2 years was 91% and 79%, respectively, in patients with preserved RAP variation and 71% and 50%, respectively, in patients with impaired RAP variation; log-rank *P* = 0.020). Moreover, preserved RAP variation was associated with a reduced univariate hazard ratio for clinical worsening (0.397 [95% confidence interval: 0.178, 0.884], *P* = 0.024). We additionally employed the 2022 European Society of Cardiology/European Respiratory Society (ESC/ERS) risk stratification scheme^1^ to assess indirectly the association of RAP variation with mortality in our cohort. As shown in Fig. 2B and Table 1, patients with preserved RAP variation had a more favourable risk distribution than those with impaired RAP variation.

### Subgroup analyses in group 1 and group 4 PH

Furthermore, subgroup analyses were conducted in patients with group 1 and group 4 PH. Table 3 presents the baseline characteristics of this subgroup. Among the 45 patients with group 1 and 4 PH, ∆RAP demonstrated a significant correlation with Eed (Spearman’s rho = − 0.529, p < 0.001). Consistent with hitherto mentioned findings in the overall study population, regression analysis indicated that only Eed, and not Ees, Ea, or tricuspid insufficiency, was associated with RV diastolic function. Moreover, ∆RAP identified high Eed with an AUROC of 0.731 [0.581, 0.882]. Survival analyses further revealed a significant association of impaired RAP variation with survival, as evidenced by Kaplan–Meier analysis (24-month survival: 37% vs 75%, log-rank p = 0.032) and Cox-regression analysis (HR 0.383 [0.154, 0.955], p = 0.039).

**Table 3:** Patient characteristics of group 1 and group 4 PH.

	n	RAP variation ≤ 2 mm Hg (n = 18)	RAP variation > 2 mm Hg (n = 27)	Combined (n = 45)	*P*
Age, years	45	67.5 [60.0, 76.0]	68.0 [51.5, 71.5]	68.0 [57.0, 76.0]	0.336^a^
Female sex, n (%)	45	11 (61)	17 (63)	28 (62)	0.900^b^
BMI, kg/m^2^	45	23.86 [23.15, 29.65]	29.37 [25.49, 33.08]	26.22 [23.47, 32.39]	0.041^a^
Group 4 PH, n (%)	45	8 (44)	12 (44)	20 (44)	1.000^b^
WHO FC, n (%)	45				0.065^b^
I		0 (0)	0 (0)	0 (0)	
II		3 (17)	7 (26)	10 (22)	
III		15 (83)	13 (48)	28 (62)	
IV		0 (0)	3 (11)	3 (7)	
NA		0 (0)	4 (15)	4 (9)	
6MWD, m	17	370 [316, 439]	402 [332, 439]	400 [317, 450]	0.962^a^
VO_2_max, mL/kg/min	24	11.40 [9.72, 12.38]	12.35 [11.38, 14.85]	12.20 [10.47, 14.15]	0.262^a^
BNP, pg/mL	44	275.5 [79.5, 402.2]	74.0 [26.0, 149.8]	99.0 [40.5, 262.2]	0.007^a^
TAPSE, mm	43	18.8 [14.3, 21.3]	21.6 [19.5, 23.7]	20.0 [16.7, 22.8]	0.006^a^
RA ESA, cm^2^	45	25.00 [19.33, 35.08]	17.20 [14.15, 19.60]	19.10 [16.20, 26.90]	< 0.001^a^
RV EDD, mm	45	51.3 ± 9.3	46.2 ± 5.4	48.2 ± 7.6	0.010^c^
TAPSE/PASP, mm/mm Hg	43	0.235 [0.183, 0.426]	0.400 [0.293, 0.572]	0.326 [0.220, 0.554]	0.024^a^
RV strain, %	45	− 14.4 ± 4.6	− 18.7 ± 2.9	− 17.0 ± 4.2	< 0.001^c^
RA reservoir strain, %	44	23.0 ± 15.8	36.5 ± 12.5	31.3 ± 15.2	0.001^c^
RA conduit strain, %	44	− 12.6 ± 8.3	− 16.5 ± 9.3	− 15.0 ± 9.0	0.218^c^
RA contractile strain, %	44	− 8.6 [− 15.4, − 2.0]	− 20.1 [− 23.6, − -14.2]	− 16.7 [− 22.8, − 9.0]	< 0.001^a^
3D RV EDV, mL	45	144.8 ± 39.6	140.6 ± 39.3	142.2 ± 39.1	0.624^c^
TI, n (%)	45				0.141^b^
None		0 (0)	4 (15)	4 (9)	
Mild		4 (22)	10 (37)	14 (31)	
Moderate		8 (45)	9 (33)	17 (38)	
Severe		4 (22)	1 (4)	5 (10)	
NA		2 (11)	3 (11)	5 (11)	
IVC diameter, mm	45	21.6 ± 5.4	17.7 ± 3.9	19.3 ± 4.9	0.013^c^
RAP, mm Hg	41	8.5 [6.0, 11.0]	6.0 [5.0, 9.0]	7.0 [5.0, 9.0]	0.105^a^
mPAP, mm Hg	45	45.0 [38.0, 49.8]	37.0 [26.5, 46.5]	39.3 [33.0, 50.0]	0.108^a^
TPR, WU	45	8.70 [7.24, 12.22]	6.67 [5.39, 10.25]	7.45 [5.81, 10.53]	0.040^a^
PAWP, mm Hg	45	10.0 [8.0, 12.5]	11.0 [7.0, 12.0]	10.0 [7.0, 12.0]	0.909^a^
Cardiac index, L/min/m^2^	45	2.45 ± 0.65	2.73 ± 0.52	2.62 ± 0.59	0.174^c^
PVR, WU	45	7.24 [4.77, 9.67]	5.29 [3.09, 7.29]	5.88 [3.51, 9.45]	0.060^a^
SvO_2_, %	45	65.1 [60.2, 66.4]	68.7 [65.8, 71.6]	66.3 [61.8, 69.8]	0.002^a^
Eed, mm Hg/mL	45	0.299 [0.199, 0.367]	0.153 [0.114, 0.235]	0.189 [0.143, 0.295]	< 0.001^a^
Ees, mm Hg/mL	45	0.822 [0.620, 0.997]	0.687 [0.529, 0.957]	0.734 [0.556, 0.989]	0.360^a^
Ea, mm Hg/mL	45	0.905 [0.604, 1.092]	0.532 [0.407, 0.846]	0.646 [0.458, 0.956]	0.021^a^
Ees/Ea	45	0.917 [0.765, 1.100]	1.116 [0.963, 1.462]	1.048 [0.902, 1.385]	0.011^a^
ESC/ERS risk, n (%)	45				0.109^b^
1		5 (28)	15 (56)	20 (44)	
2		10 (56)	11 (41)	21 (47)	
3		3 (17)	1 (4)	4 (9)	
S′/RAAi, m^2^/(s cm)	45	0.781 ± 0.362	1.393 ± 0.569	1.148 ± 0.578	< 0.001^c^
Respiratory RAP variation, mm Hg	45	1.0 [0.7, 1.2]	5.3 [3.4, 6.4]	3.0 [1.0, 5.7]	< 0.001^a^

Data are presented as n (%) for categorical variables, mean ± standard deviation for normally distributed continuous variables, and median [Q1, Q3] for non-normally distributed continuous variables.

6MWD, 6-min walk distance; BMI,  body mass index; BNP,  brain natriuretic peptide; Ea,  arterial elastance; EDD,  end-diastolic diameter; EDV,  end-diastolic volume; Eed,  end-diastolic elastance; Ees,  end-systolic elastance; ESA, end-systolic area; ESC/ERS,  European Society of Cardiology/European Respiratory Society; IVC,  inferior vena cava; mPAP,  mean pulmonary artery pressure; NA,  not available; PASP,  pulmonary artery systolic pressure; PAWP, pulmonary artery wedge pressure; PVR,  pulmonary vascular resistance; RA,  right atrial; RAP,  right atrial pressure; RV,  right ventricular; S′/RAAi,  ratio of peak lateral tricuspid annulus systolic velocity to right atrial area index; SvO_2_,  mixed venous oxygen saturation; TAPSE,  tricuspid annular plane systolic excursion; TI , tricuspid insufficiency; TPR,  total pulmonary resistance; VO_2_max ,  maximum oxygen uptake; WHO FC,  World Health Organization functional class; WU,  Wood Units.

^a^Wilcoxon test.

^b^Chi-square test.

^c^T-test.

## Discussion

In this study, we demonstrate that the variation of RAP during spontaneous respiration serves as a surrogate of RV diastolic function, is independent from RV-PA coupling or the severity of tricuspid regurgitation, and predicts diastolic reserve and cardiac output/index increase during exertion. Furthermore, we validate its prognostic significance concerning clinical worsening and estimated risk of mortality. The study was conducted using gold-standard conductance catheterization to assess RV diastolic function and RV-PA coupling.

Our study is the first to invasively validate the association between respiratory variation of RAP during spontaneous breathing and RV diastolic function based on the end-diastolic pressure–volume relationship. Notably, RAP variation was not independently associated with other signs of RV maladaptation, such as RV-PA uncoupling or tricuspid regurgitation severity. This observation is consistent with previous findings indicating that cardiac output/index does not differ according to RAP variation at rest^4^. The correlation observed between RAP variation and RV diastolic performance in our study highlights the potential of RAP variation to mirror the complex mechanisms of RV relaxation and filling during diastole. This extends our understanding beyond conventional measurements since RAP itself was not significantly associated with Eed in our study population. The simplicity of the assessment of RAP variation increases the relevance of this finding, as it can be routinely monitored during right heart catheterization.

ROC analyses indicated that RAP variation also has good discriminatory power for the differentiation of preserved and impaired RV diastolic function. Considering the invasiveness and complexity of assessment of RV diastolic function, RAP variation can serve as a simple surrogate for this parameter. Recently, we derived and validated (independently in a second cohort) the ratio of peak lateral tricuspid annulus systolic velocity to RA area index as a novel and first echocardiographic indicator of RV diastolic function^10^. Though numerically the area under the ROC curve for RAP variation was lower than the one described for the aforementioned echocardiographic ratio^10^, RAP variation gives additional information based on routine right heart catheterization.

Interestingly, there were meaningful differences between patients with impaired and preserved RAP variation during exertion. While contractile reserve was comparable between the two groups, patients with impaired RAP variation showed more prominent RV dilatation during exercise, which is known to be associated with clinical worsening^11,12^. Consistent with this finding, patients with impaired RAP variation also had a significantly greater increase of Eed than those with preserved RAP variation, indicating an impaired diastolic reserve in the former group, which results in significantly reduced cardiac output and cardiac index during exercise. Thus, impairment of RAP variation during spontaneous breathing is not only an indicator for RV diastolic function at rest but is also associated with exercise haemodynamics.

Al-Qadi et al.^4^ showed in a retrospective study the prognostic relevance of RAP variation during spontaneous breathing. In line with their observations, our study shows significant associations with clinical worsening as well as ESC/ERS risk scoring. The ESC/ERS risk stratification scheme is the current gold standard in Europe to estimate 1-year survival prognosis and was originally developed for patients with group 1 PH, but is also validated in severe interstitial lung disease-associated PH and chronic thromboembolic PH^1,13–16^. Since the risk scheme is highly validated, impaired RAP variation is likely to be associated with hard endpoints such as death^17,18^. This is consistent with previous research showing numerically higher (though statistically not significant) 1-year mortality in patients with impaired RAP variation compared with those with preserved RAP variation^4^.

Furthermore, studies have demonstrated that intrathoracic pressure, as measured via esophageal manometry, exerts a significant impact on pulmonary hemodynamics, particularly on static values in obese patients^19^. While its influence on respiratory variation is presumed to be less pronounced, additional research investigating the relevance of intrathoracic pressure on ∆RAP is warranted.

Our study is limited by its retrospective study design and lack of long-term survival data. The inclusion of only one centre in the study may limit the generalizability of the results. Nonetheless, implementation of highly sophisticated hemodynamic data and pressure–volume loops using gold-standard conductance catheterization at rest and during exercise shed light on the pathophysiological mechanism behind the impairment of RAP variation in patients with PH.

### Interpretation

**Figure 3:**
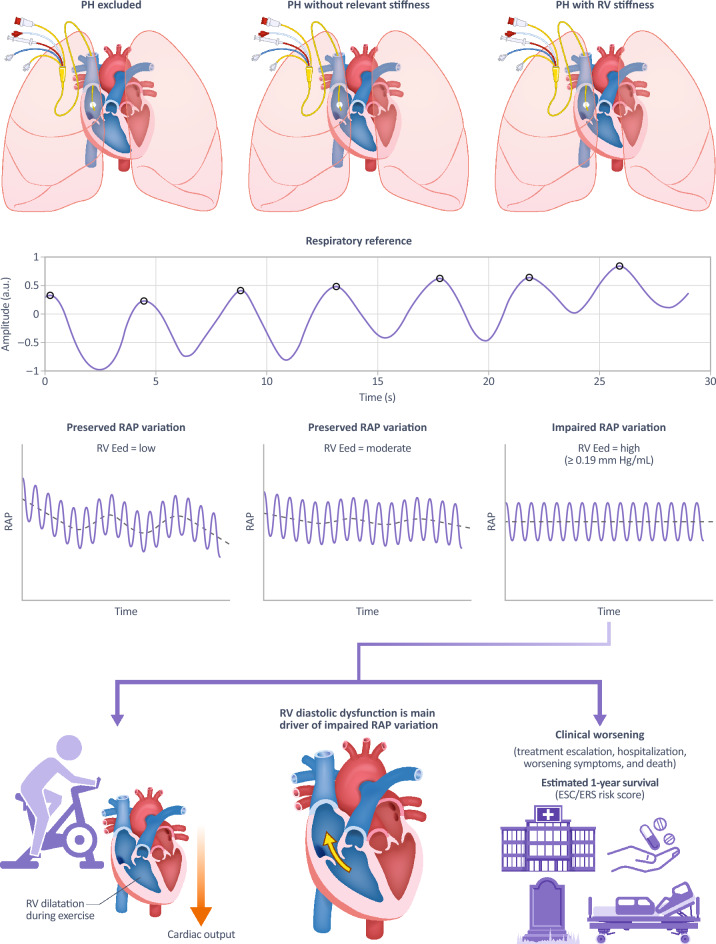
Overview of key findings. Respiratory RAP variation in patients with PH primarily hinges on diastolic RV function. Dilatation during exercise and subsequently reduced peak cardiac output occurs more often in patients with impaired RAP variation than in those with preserved RAP variation. Lastly, impaired variation of RAP is linked with clinical worsening and a well-established surrogate of mortality. A. u., arbitrary units; Eed, end-diastolic elastance; PH,  pulmonary hypertension; RAP,  right atrial pressure; RV,  right ventricular.

RAP variation in patients with PH primarily hinges on diastolic RV function rather than systolic RV function, RV-PA coupling or tricuspid regurgitation (Fig. 3). RV dilatation during exercise with impaired diastolic reserve and subsequently reduced peak cardiac output occurs more often in patients with impaired RAP variation than in those with preserved RAP variation. Lastly, impaired variation of RAP is linked with clinical worsening and risk scores indicating increased mortality.

## Data Availability

The datasets analysed during the current study are available from the corresponding author on reasonable request.

## Abbreviations

6MWD: 6-Minute walk distance
ΔRAP: Disparity between end-expiratory and end-inspiratory right atrial pressure
Ea: Arterial elastance
EDV: End-diastolic volume
Eed: End-diastolic elastance
Ees: End-systolic elastance
ESC/ERS: European Society of Cardiology/European Respiratory Society
PA: Pulmonary arterial
PH: Pulmonary hypertension
RA: Right atrial
RAP: Right atrial pressure
ROC: Receiver operating characteristic
RV: Right ventricular
WHO: World Health Organization
